# Pilot-Scale Polysulfone Ultrafiltration Patterned Membranes: Phase-Inversion Parametric Optimization on a Roll-to-Roll Casting System

**DOI:** 10.3390/membranes15080228

**Published:** 2025-07-31

**Authors:** Ayesha Ilyas, Ivo F. J. Vankelecom

**Affiliations:** Membrane Technology Group (MTG), Division cMACS, Faculty of Bioscience Engineering, KU Leuven, Celestijnenlaan 200F, P.O. Box 2454, 3001 Leuven, Belgium; ayesha.ilyas@kuleuven.be

**Keywords:** patterned membranes, phase inversion, pilot-scale preparation, fouling control, roll-to-roll membrane production, upscaling

## Abstract

The scalability and processability of high-performance membranes remain significant challenges in membrane technology. This work focuses on optimizing the pilot-scale production of patterned polysulfone (PSf) ultrafiltration membranes using the spray-modified non-solvent-induced phase separation (s-NIPS) method on a roll-to-roll pilot line. s-NIPS has already been studied extensively at lab-scale to prepare patterned membranes for various applications including membrane bioreactors (MBR), reverse osmosis (RO) and forward osmosis (FO). Although studied at the lab scale, membranes prepared at a larger scale can significantly differ in performance; therefore, phase inversion parameters, including polymer concentration, molecular weight, and additive type (i.e., polyethylene glycol (PEG) or polyvinylpyrolidine (PVP)) and concentration, were systematically varied when casting on a roll-to-roll, 12″ wide pilot line to identify optimal conditions for achieving defect-free, high-performance, patterned PSf membranes. The membranes were characterized for their pure water permeance, BSA rejection, casting solution viscosities, and resulting morphology. s-NIPS patterned membranes exhibit 150–350% increase in water flux as compared to their reference flat membrane, thanks to very high pattern heights up to 825 µm and formation of finger-like macrovoids. This work bridges the gap between lab-scale and pilot-scale membrane preparation, while proposing an upscaled membrane with great potential for use in water treatment.

## 1. Introduction

Membrane technology, with its advantages of lower footprint, low energy consumption, high selectivity, reduced waste production, and high versatility, is widely used in industrial processes. Recent work on membrane development has largely focused on using new membrane materials and methods for emerging applications or to tackle major issues like fouling [1,2,3,4,5,6]. However, the majority of these membranes are prepared at the lab scale and yield excellent performance during lab evaluations, they face significant challenges in terms of scalability, handling, and processability for future production and large-scale use.

Commercial membrane manufacturing often involves the phase inversion of polymer solutions, induced by either immersing a cast film in a non-solvent (non-solvent-induced phase separation, NIPS) or by changing the temperature, introducing non-solvent vapors, or solvent evaporation [7,8]. NIPS is the main production process for microfiltration (MF), ultrafiltration (UF), and nanofiltration (NF) membranes, as well as for producing porous supports for reverse osmosis (RO) and gas separation (GS) membranes. NIPS enables the continuous roll-to-roll production of membranes with properties governed by various phase-inversion parameters [9].

Given the advantages and simplicity of NIPS, spray-modified non-solvent-induced phase separation (s-NIPS) was introduced in 2020 by Vankelecom et al. [10]. s-NIPS is a modification of the conventional NIPS process where a limited amount of non-solvent is sprayed to induce coagulation. The spray was used to solidify the surface patterns created by a 3D-printed patterned casting knife to prepare a patterned membrane [11]. s-NIPS patterned membranes have shown great potential in mitigating fouling and increasing membrane permeances as compared to corresponding conventional flat membranes [12]. Patterns up to 750 ± 100 µm could be created, and the resulting membranes showed a 70–300% decrease in the membrane fouling rate and a doubled critical flux compared to the flat counterpart during wastewater treatment. Vortex formation and the additional surface area of the patterned membrane help minimize membrane fouling and delay its onset [13,14]. Similar to NIPS, s-NIPS enables the continuous production of membranes with properties controlled by the choice of polymer and additives, their concentration, the type of solvent and non-solvent, and the casting conditions [15]. Therefore, s-NIPS membranes show great potential for replacing conventional flat membranes for various applications, offering a high effective membrane area per module volume and increased anti-fouling properties.

The majority of the literature studies have reported optimization of flat-sheet PSf membranes via the conventional NIPS process realized by immersing the cast polymer solution into a non-solvent bath. In this work, the large-scale production of patterned PSf membranes was optimized for the first time on a 30 cm wide roll-to-roll (R2R) Smartcoater-300 pilot line via the s-NIPS process to enable the future continuous production of these membranes for various applications, such as membrane bioreactors (MBRs) and UF support for RO/NF [12,14]. The suggested modifications can be applied to any industrial R2R system for the continuous production of these membranes. Phase inversion parameters that could affect the membrane morphology and pattern homogeneity were investigated in detail to provide guidelines for the pilot-scale fabrication of patterned PSf membranes. The polymer concentration, polymer molecular weight, choice of additive (i.e., polyethylene glycol (PEG) or polyvinylpyrrolidone (PVP)), and their molecular weight and concentration were systematically varied to identify the optimal casting conditions to achieve high permeance, good rejection, and homogenous patterning. Overall, this study explored the potential for the commercial-scale mass production of PSf patterned membranes.

## 2. Materials and Methods

### 2.1. Dope Solution Preparation

To prepare the polymer solutions, PSf pellets were dried in an oven at 100 °C for 24 h. PVP (10 kDa, Sigma Aldrich, Hoeilaart, Belgium) and PEG (10 kDa or 20 kDa, Sigma Aldrich, Hoeilaart, Belgium) were dissolved in N-methyl-2-pyrrolidone (NMP, 99 wt%, Acros Organics, Geel, Belgium). The casting solutions were stirred at 180 rpm at 60 °C for 24 h and then degassed overnight. Based on previous experience with s-NIPS-patterned membranes on a lab scale, high polymer and additive concentrations were used to achieve high casting solution viscosities. The PSf concentrations in the dope solution varied between 18 and 22 wt%, while the additive concentration was kept at 10wt%, 15wt% and 20 wt% for each polymer concentration. PSf with different molecular weights (MW), i.e., Ultrason^®^ S6010 (61 kDa, BASF, Ludwigshafen, Germany) and Udel^®^ P-3500 (75 kDa, Solvay, Brussels, Belgium) were used.

### 2.2. Membrane Characterizations

Filtrations were performed using a high-throughput dead-end filtration system to obtain the pure water permeance (PWP) of the membranes [16]. For each membrane, a minimum of four coupons (d_eff_ = 14 mm) were tested from at least five batches of membranes. The pressure was maintained at 3 bar throughout the process. Measurements were taken at steady state, which was assumed when a variance of less than 5% between successive measurements of permeate flux was obtained for a coupon. PWP (L m^−2^ h^−1^ bar^−1^) was calculated as follows:

*PWP* = *V*/*TMP* × *A*_m_ × *t*

where TMP is the transmembrane pressure (bar), A_m_ is the membrane surface area (m^2^), V is the permeate volume (L), and t is the filtration time (h).

Membrane rejection was determined via filtration with a 1 g/L solution of bovine serum albumin (BSA, 66 kDa, 98%, Sigma Aldrich, Hoeilaart, Belgium) in DI water stirred for 3 h prior to filtration. Stabilization with DI water was performed for 3 h while the pressure was maintained at 3 bar. Sequentially, the BSA solution was filtered, and the occurrence of concentration polarization was minimized via continuous stirring of the BSA solution during the process. The concentrations of BSA in the permeate (C_p_) and feed (C_f_) were determined using a UV-VIS spectrophotometer (Shimadzu UV-1800, Kyoto, Japan) by measuring the absorbance at 278 nm. Rejections were calculated as follows:

*R* (%) = (1 − *C*_*p*_/*C*_*f*_) × 100

Membrane morphology and pattern homogeneity were observed using scanning electron microscopy (SEM, JEOL JSM-6010LV, Tokyo, Japan). The membrans’ morphology was preserved by immersion in liquid N_2_ while breaking the membranes over a sharp knife to create cross-sectional samples. Subsequently, the samples were placed on an SEM holder and coated with a gold-palladium layer using an Auto Fine Coater (JFC-1300, Tokyo, Japan). Furthermore, image analysis was performed using the InTouch imaging software (JEOL). The membrane was dimensioned using ImageJ (version 1.53K, NIH, Bethesda, MD, USA).

Viscosity (mPa·s) measurements were performed at room temperature using a rotational viscometer equipped with a modular Visco QC300 (Anton Paar, Graz, Austria) operating within the range of 10 to 100 s^−1^ steady-state shear rate. The reported values are the averages of two measurements taken at a shear rate of 10 s^−1^.

The bulk porosity ε (%) of the membranes was determined using a gravimetric method. The calculation was performed using the following equation, where the total pore volume is assumed to be filled with liquid and is divided by the volume of the membrane:

*ε* (%) = *w*_*w*_ − *w*_*d*_/*A* × *l* × *ρ*

where w_w_ is the weight of the wet membrane weighed after removing excess water with filter paper (g), w_d_ is the weight of the membrane dried at 60 °C for 24 h in an air-circulating oven (g), A is the effective surface area of the membranes (cm^2^), and ρ is the density of pure water (0.998 g/cm^3^). An average of three samples was collected for each membrane.

Thermogravimetric analysis (TGA) measurements (STA 449, NETZSCH, Selb, Germany) were performed for the pristine polymer, additives, and membranes to gravimetrically quantify the amount of additive within the membrane. Around ±5 mg of the sample was placed in an 85 µL alumina crucible. Afterwards, the sample was heated at a rate of 5 °C/min until 700 °C under an O_2_ atmosphere, and the residual mass was measured using a high-resolution (0.1 µg) balance system.

### 2.3. Roll-to-Roll (R2R) System Design

**Figure 1:**
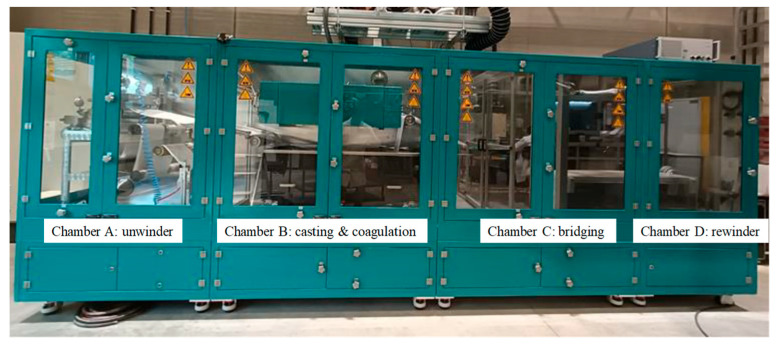
The four chambers of KU Leuven R2R pilot system with unwinder in chamber A, a casting and coagulation chamber B, chamber C merely used in current configuration to bridge the distance to the rewinder in chamber D.

Membranes at pilot-scale were prepared using the R2R system (Smartcoater-300, Coatema^®^, Dormagen, Germany), as shown in Figure 1. This KU Leuven system provides various pre- or post-treatment possibilities and enables the production of 30 cm wide flat-sheet membranes in an R2R layout. The system has an adaptable layout consisting of casting, coagulation, and post-treatment chambers, along with an unwinder and rewinder. These chambers can be configured and modified with additional technologies according to the fabrication process. A non-woven support of polypropylene/polyethylene (PP/PE) (Novatexx 2471, Freudenberg, Germany) was used, and its path was adjusted via guiding rollers. The tension and speed rollers precisely measure and regulate the tension and moving speed throughout the process.

**Figure 2:**
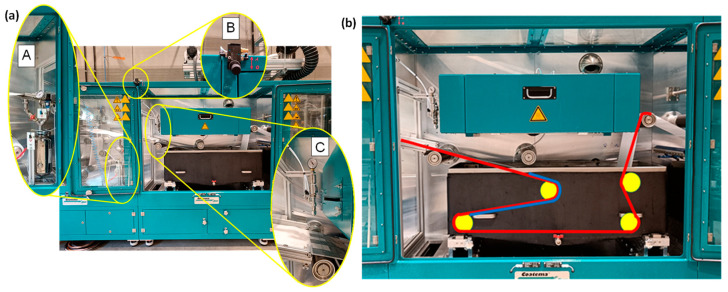
(**a**) Overview of the new installations on the pilot line, which included a pressurized water-containing vessel (**A**), a controller for the sprayer (**B**) and a sprayer above the casting platform (**C**). (**b**) The path that the membrane will follow is highlighted as red, while the blue line shows the 70 cm distance over which the patterned surface of the membrane will not touch the guiding rollers.

At the lab-scale, patterned membranes were prepared via s-NIPS, which required an automated sprayer (AutoJet^®^ Model 2008+, Spraying Systems Co.^®^, Deinze, Belgium) and a 3D-printed, patterned casting knife [7,10]. Therefore, some adjustments had to be made to the pilot system to prepare R2R patterned membranes. As described in a previous study [17], the major modification included the installation of a casting platform mounted on an aluminum framework along with an air-actuated hydraulic nozzle-type sprayer (AA22AUH-SS-14799, Spraying Systems Co.^®^) above the casting platform in chamber B of the pilot line (Figure 1 and Figure 2a). This guaranteed direct spraying immediately after casting the membrane and provided the shortest time between membrane casting and immersion in the coagulation bath. Spraying with a non-solvent ensures the fixing of the surface patterns as early as possible and prevents the re-flow of the patterns that would otherwise relapse on the still liquid cast film. Subsequently, the partially solidified film was immersed in a 180 L coagulation bath for further membrane solidification over the full depth. Figure 2a shows the 5 L vessel installed to store and pressurize the sprayed water, as well as the controller for the (vertically and horizontally) adjustable sprayer above the casting platform. The choice of spray nozzle was identified as a crucial factor when installing the sprayer. The spray nozzle controls the amount of water that can be sprayed on top of the wet membrane to form a film of water that allows the fast fixation of the membrane patterns. Nozzle tip UniJet 95015 was used for its high water flow rate (i.e., 700 mL/min at 6 bar). More details on the various flow nozzles can be found in a previous report [17].

**Figure 3:**
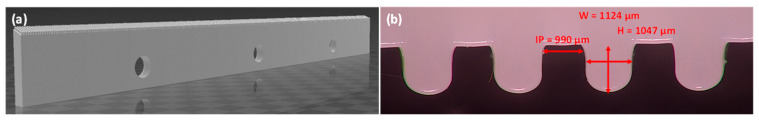
(**a**) 3D model and (**b**) light microscopy image of printed patterned casting knife.

During casting, the non-woven was arranged on the guiding rollers to form a path such that the patterns created on top of the membranes avoided touching the rollers for as long as possible. This creates a length of about 70 cm, as indicated by the blue line in Figure 2b. A 30 cm wide rectangular-patterned casting knife was printed using an Objet30 printer (Stratasys Ltd., Eden Prairie, MN, USA) and attached to a conventional casting knife. Rectangular pattern teeth were designed 500 µm apart from each other, with each measuring 1000 µm in height and 500 µm in width. Figure 3 shows the designed 3D model and light microscopy image of the final printed patterned casting knife.

## 3. Results

### 3.1. Patterned Membrane Preparation

**Figure 4:**
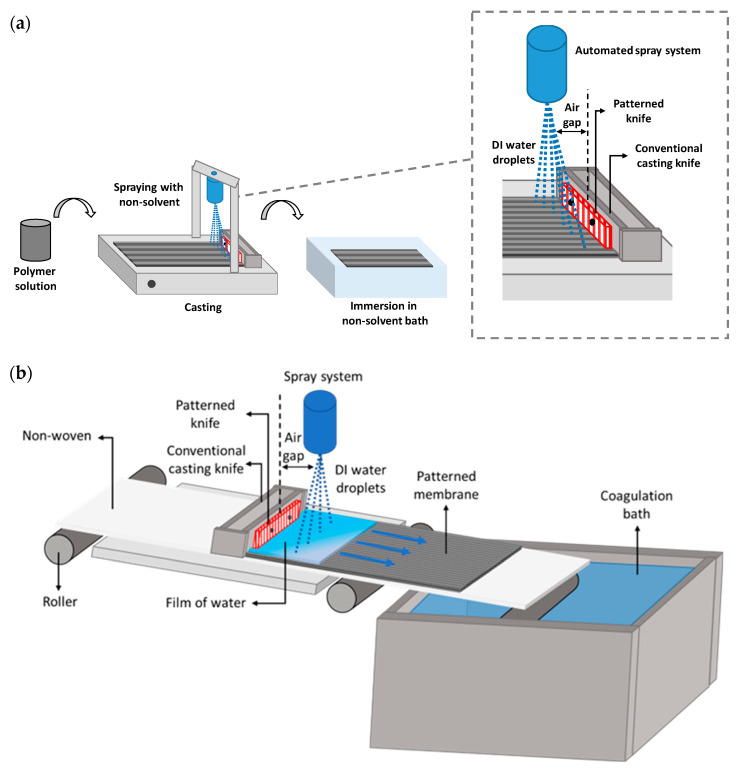
Scheme of the patterned membrane preparation (**a**) at lab-scale and (**b**) its installation on a pilot-scale roll-to-roll system.

The casting procedures at the pilot and lab scales are distinguished in terms of their dynamics. At the lab-scale, the casting knife moves on top of the stationary non-woven fixed to a glass plate (Figure 4a), while in the R2R system, the casting knife remains stationary and the non-woven moves underneath it (Figure 4b). This implies that the dope solution is carried along by the moving non-woven underneath the casting knife rather than being distributed by the displacement of the casting knife itself. Moreover, the absence of the glass plate in the R2R system alters the phase inversion dynamics due to the out-diffusion of the solvent and in-diffusion of water from both the top and bottom of the membrane. In addition, the time between casting and entering the coagulation bath is different in both systems, thus impacting the penetration of the cast polymer solution into the non-woven and the contact with the environment.

The casting speed for patterned membranes at the lab scale was maintained at 2.5 m/min. However, visible defects were formed when the same casting speed was used in the R2R system. This could be the result of an uneven casting solution distribution due to the high speed at which the non-woven fabric moved underneath the casting knife. Moreover, to avoid the blockage of the casting knife due to early polymer coagulation underneath the knife, the casting speed was adjusted to 1 m/min, which resulted in a visibly defect-free membrane. The sprayer height was fixed at 12 cm to cover the spraying width of 30 cm, casting thickness was set at 200 µm, and the sprayed water flow rate was 700 mL/min at 6 bar. These casting parameters formed a sprayed water layer of about 2 mm thickness and a coagulation time of 2 min, which was sufficient for the early precipitation of the patterns and subsequent complete film formation for PSf [17]. Subsequently, the membrane was removed from the rewinder in the last chamber and stored as needed.

### 3.2. Performance Comparison for PEG and PVP-Based Membranes

**Figure 5:**
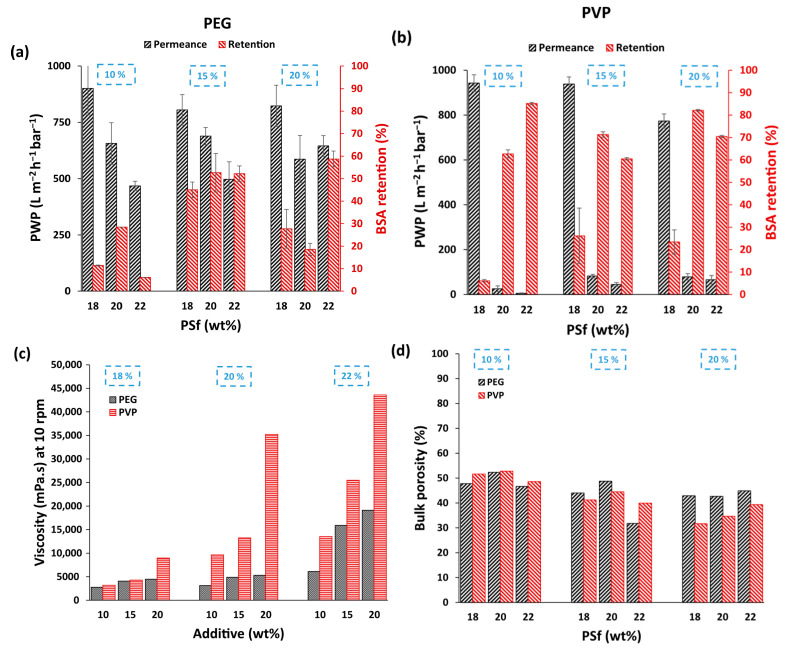
PWP and BSA retentions of patterned membranes prepared with PSf concentration from 18–22 wt% with 10, 15 and 20 wt% (**a**) PEG and (**b**) PVP. (**c**,**d**) show the comparison of casting solution viscosity and bulk porosity of cast membranes, respectively, for all PEG and PVP containing membranes.

**Figure 6:**
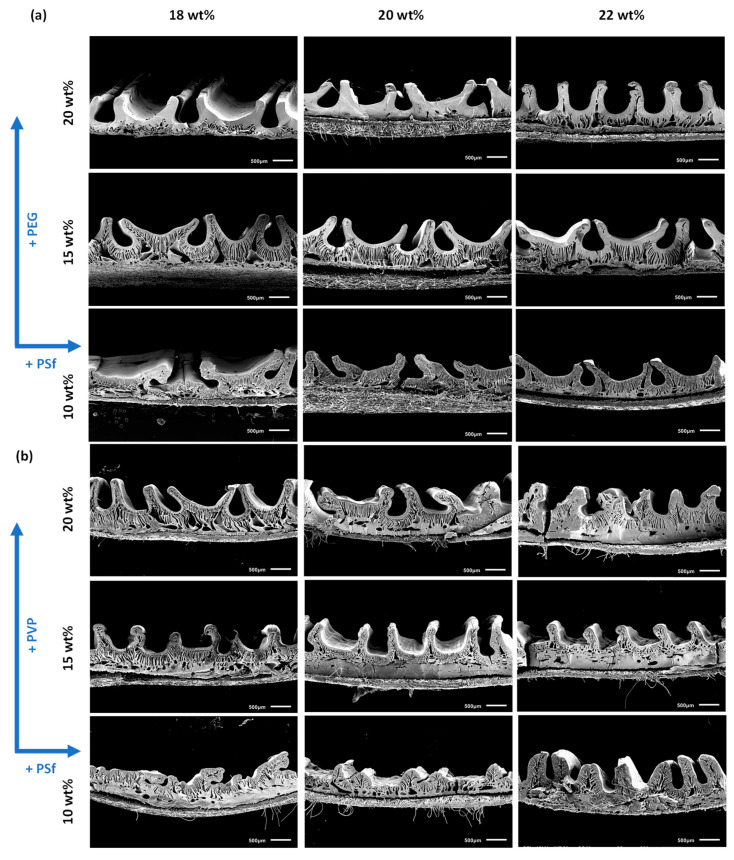
SEM images of patterned membranes with 18–22 wt% PSf concentration and 10–20 wt% of (**a**) PEG and (**b**) PVP.

In order to evaluate the effect of hydrophilic additives, i.e., PEG and PVP, on the performance of PSf patterned membranes, casting solutions were prepared with 10 wt%, 15 wt% and 20 wt% concentrations of both additives with similar MW (i.e., 10 kDa) in solutions containing 18 wt%, 20 wt% and 22 wt% PSf dissolved in NMP. Figure 5 shows a comparison of the performances of the membranes prepared with PEG (Figure 5a) and PVP (Figure 5b). As expected, a decrease in permeance and improved retention were generally observed with an increase in polymer concentration when the additive concentration was kept constant. This was due to the increased solution viscosities that tend to slow down phase inversion, resulting in relatively denser membranes [16]. Cross-sectional SEM images (Figure 6a,b) also showed thicker, dense top layers with an increase in polymer concentration, regardless of the additive used. In addition, the patterns became slightly higher, which can help to increase the permeance due to the increase in the effective surface area. In contrast, both retention and permeance increased with an increase in the concentration of both additives for similar polymer concentrations. These observations are highly dependent on the MW and concentration of the additives. At lower concentrations, the additives act as pore formers due to their non-solvent character, resulting in thermodynamically less stable casting solutions, leading to increased pore sizes and porosities [18,19]. However, at higher concentrations, these additives can also act as macrovoid suppressors, resulting in denser membranes [20]. This is related to the increase in solution viscosity (Figure 5c), where solution demixing is delayed as the rheological impact overcomes the thermodynamic enhancement. For corrugated membranes, however, higher additive concentrations can also result in more pronounced and homogeneous patterning due to the combined effect of thermodynamic enhancement and increased solution viscosity, which is sufficient to achieve good retention of the patterns, thereby increasing their PWP [15]. Unlike flat membranes, the increase in permeance here is also correlated with the increase in surface area due to both improved pattern formation at higher viscosities and increased hydrophilicity associated with more additives that are left behind in the membranes.

Comparing Figure 5a,b, lower permeances were observed with improved retention when PVP was used as an additive compared to PEG-based membranes. The SEM images in Figure 6b also show relatively denser membranes and more homogenous patterning than those in Figure 6a for PEG-based membranes, thereby explaining the improved retentions and lower permeances. A comparison of the casting solution viscosities (Figure 5c) showed higher solution viscosities for all PVP-based membranes. This was an interesting observation because both additives have similar MWs with similar polymer and additive concentrations. The higher viscosity of the PVP-based solutions was hypothesized to be due to stronger interactions and solvation between PVP and NMP in contrast to PEG, which was also evident when their respective distances in Hansen space (R_a_) were compared (Table 1) [21,22,23,24]. The comparison of the bulk porosities in Figure 5d clearly shows higher porosities for PEG-based membranes, with the difference becoming more evident as the additive concentration increased. These observations support the hypothesis that the higher viscosity of PVP-containing casting solutions creates a higher kinetic hindrance during phase inversion, leading to denser membranes with better pattern preservation [21].

As the study employed relatively high concentrations of additives, TGA was performed in order to quantify the amount of residual PEG and PVP that could further support the experimental observations. Table 2 shows the mass loss (%) calculated for both PEG- and PVP-based membranes until the additives were fully degraded. Both PEG and PVP were retained in the membrane matrix, with PVP in higher amounts, which supported the hypothesis of enhanced PVP-NMP interactions and, thereby, higher residual PVP. This also explains the lower PWP and higher retention observed for the PVP-based membranes (Figure 5b).

Although PVP-containing membranes resulted in much better patterns and retentions, the membranes became extremely rigid and prone to defects during handling. Therefore, the following membranes were prepared using PEG.

### 3.3. Effect of Polymer MW

**Figure 7:**
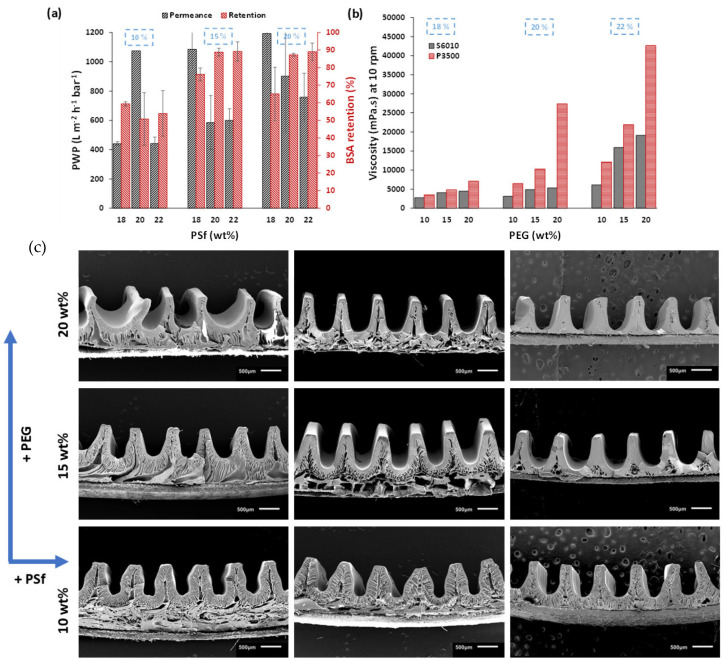
(**a**) PWP and BSA retentions, (**b**) comparison of viscosity of casting solutions prepared with S6010 and P3500 PSf and (**c**) cross-sectional images of patterned membranes prepared with P3500 PSf concentrations from 18–22 wt% with 10, 15 and 20 wt% PEG.

Because the polymer MW affects the viscosity of the casting solution, PSf with a higher MW was investigated using PEG. PSf S6010 (MW 61 kDa) and Udel P3500 (MW 75 kDa) were used [25]. Casting solutions were prepared with 10 wt%, 15 wt% and 20 wt% concentrations of PEG in solutions containing 18 wt%, 20 wt% or 22 wt% PSf dissolved in NMP. Membranes were cast at 0.1 m/min for reasons of pattern homogeneity since lower speeds increase the amount of water sprayed. This also changed the coagulation time to 20 min. Figure 7a shows the improved retention and permeance when high MW PSf was used to prepare the membranes. Figure 7b also shows the higher casting solution viscosity for these high-MW PSf solutions. The SEM images showed homogenous patterns with a high pattern height fidelity between 70 ± 2% and 80 ± 2%. Membranes with 15 wt% PEG generally exhibited greater homogeneity over larger membrane sheets. Table 3 shows the performance characteristics of the best membranes, where the permeances varied between 1085 ± 124 L m^−2^ h^−1^ bar^−1^ and 598 ± 80 L m^−2^ h^−1^ bar^−1^ with pattern heights varying between 725 ± 25 and 825 ± 15 µm. It is important to note that the PWP was calculated based on the actual area of the patterned membranes (based on the pattern dimensions observed via SEM). However, these values are higher than those of the reference flat membranes prepared with a similar composition. This supports the hypothesis mentioned in previous studies [15] that the observed increase in permeance for patterned membranes is not only due to the higher effective surface area but also due to the morphological changes induced by the modified phase inversion, i.e., s-NIPS.

### 3.4. Effect of PEG MW

**Figure 8:**
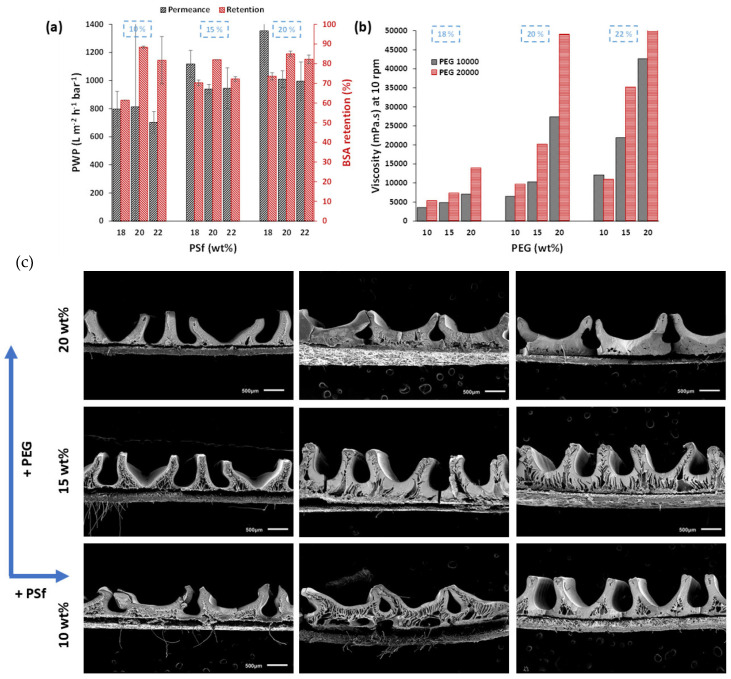
(**a**) PWP and BSA retentions, (**b**) comparison of viscosity of casting solutions prepared with 10 kDa and 20 kDa PEG, and (**c**) cross-sectional images of patterned membranes prepared with P3500 PSf concentration from 18–22 wt% with 10, 15 or 20 wt% 20 kDa PEG.

Although PEG (MW: 10 kDa) with P3500 PSf resulted in homogeneously patterned membranes with excellent performance, using PEG with a higher MW could result in further increased viscosities to improve the pattern fidelity and homogeneity. Membranes were thus also prepared using 20 kDa PEG. As anticipated, Figure 8 shows the higher viscosities of the casting solutions when PEG 20 kDa was used, as a longer PEG chain length results in stronger entanglement with PSf chains and more interactions per PEG chain with the solvent [26]. This resulted in a morphological change for 20 kDa PEG-based membranes from finger-like macrovoids for 10 kDa PEG-based membranes to dense sponge-like structures. Increased viscosity reduces the precipitation rate during phase inversion [26]. An increase in PEG MW resulted in membranes with higher PWP and slightly lower BSA retention. For the 20 wt% PSf–15 wt% PEG membrane, the PWP increased from 585 ± 103 L m^−2^ h^−1^ bar^−1^ to 938 ± 33 L m^−2^ h^−1^ bar^−1^, and the retention decreased from 88 ± 2% to 82 ± 1%. Higher additive retentions, as observed in Table 4, when using 20 k PEG, rendered the membrane more hydrophilic and therefore resulted in increased PWP. However, the pattern height fidelity reduced to 58%, and the homogeneity was significantly compromised, as shown in Figure 8c. This is mainly because the increased viscosities led to higher kinetic hindrance during the NIPS process. This lengthens the time scale for pattern solidification, leading to a loss in the final pattern dimensions and shape. Therefore, PEG with a 10 kDa MW was found to be more suitable for achieving patterns with good homogeneity and high performance with minimal defects (Table 3).

## 4. Discussion

The pilot-scale production of patterned PSf UF membranes was successfully optimized using the s-NIPS method in a roll-to-roll coating system. Defect-free and homogeneously patterned PSf membranes were prepared by varying the polymer concentration, polymer MW, choice of hydrophilic additive (PEG or PVP), and its concentration and MW. Although PVP addition resulted in membranes with good rejection and better pattern homogeneity, their high rigidity led to defect formation during handling. Therefore, 15–20 wt% PEG was used with longer chain PSf, which significantly improved the pattern homogeneity and pattern height fidelity, i.e., 85% with a high PWP (1085 ± 124 L m^−2^ h^−1^ bar^−1^) and good BSA rejections (90 ± 3%). Further increasing the MW of PEG resulted in a further increase in the water flux with similar BSA rejections; however, the pattern height fidelity substantially dropped to 58%, and the pattern formation was significantly less homogeneous over the membrane, mainly due to a more delayed phase inversion as the casting solution viscosities increased. Overall, this work offers a scalable and efficient method for the continuous production of high-performance UF membranes. An appropriate casting platform, well-calibrated casting knife, homogenous solution preparation, slow casting speed, and sufficient non-solvent flow rate while spraying were the key parameters that ensured good batch reproducibility of these membranes. These advancements pave the way for future industrial-scale production of patterned membranes, addressing the scalability and processability challenges in membrane technology, and allowing the creation of high-flux/high-rejection UF membranes with potentially reduced fouling propensity, which will be investigated in the near future.

## Figures and Tables

**Table 1:** Distance in Hansen space (R_a_) of PEG and PVP with NMP based on their Hansen solubility parameters.

Component	δ_D_	δ_P_	δ_H_	R_a_ (MPa^1/2^)
NMP	18.0	12.3	7.2	-
PEG	15.4	7.6	8.7	7.1
PVP	15.5	11.7	8.6	5.2

**Table 2:** Mass loss observed during TGA for membranes with 22 wt% PSf and additive concentration varying between 10–22 wt%.

Additive Concentration (wt%)	PEG Mass Loss (%)	PVP Mass Loss (%)
10	17.1	31.0
15	17.3	32.5
20	17.3	33.7

**Table 3:** Performance characteristics of the final selected patterned membranes prepared with P3500 PSf concentration from 18–20 wt% and 15 wt% PEG.

Membrane (PSf-PEG)	Pattern Height * (µm)	Surface Area Increase (%)	PWP_flat_ Membrane (L m^−2^ h^−1^ bar^−1^)	PWP_patterned_ Based on Real Area * (L m^−2^ h^−1^ bar^−1^)	PWP_patterned_ Based on Projected Area (L m^−2^ h^−1^ bar^−1^)	PEG Mass Loss (%)
18–15	725 ± 25	175 ± 17	239 ± 24	351 ± 39	1085 ± 124	20
20–15	783 ± 28	198 ± 16	219 ± 19	345 ± 32	585 ± 103	20
22–15	825 ± 15	200 ± 13	223 ± 17	236 ± 29	598± 90	22

* SEM-based.

**Table 4:** Mass loss observed during TGA for membranes with 22 wt% PSf and 20 kDa PEG concentration varying between 10–20 wt%.

Additive Concentration (wt%)	PEG Mass Loss (%)
10	27.1
15	27.1
20	27.3

## Data Availability

Dataset available on request from the authors.
